# Pre-Treatment with Grape Seed Extract Reduces Inflammatory Response and Oxidative Stress Induced by *Helicobacter pylori* Infection in Human Gastric Epithelial Cells

**DOI:** 10.3390/antiox10060943

**Published:** 2021-06-11

**Authors:** Jose Manuel Silvan, Alba Gutierrez-Docio, Esperanza Guerrero-Hurtado, Lucia Domingo-Serrano, Ana Blanco-Suarez, Marin Prodanov, Teresa Alarcon-Cavero, Adolfo J. Martinez-Rodriguez

**Affiliations:** 1 Microbiology and Food Biocatalysis Group (MICROBIO), Department of Biotechnology and Food Microbiology, Institute of Food Science Research (CIAL, CSIC-UAM), C/Nicolás Cabrera, 9. Campus de Cantoblanco, Universidad Autonoma de Madrid, 28049 Madrid, Spain; luciadomingoserrano@gmail.com; 2 Department of Production and Characterization of Novel Foods, Institute of Food Science Research (CIAL, CSIC-UAM), C/Nicolas Cabrera 9, Campus de Cantoblanco, Universidad Autonoma de Madrid, 28049 Madrid, Spain; alba.gutierrez@uam.es (A.G.-D.); esperanza.guerrero@estudiante.uam.es (E.G.-H.); marin.prodanov@uam.es (M.P.); 3 Microbiology Department, Sanitaria Princesa Research Institute, Hospital Universitario de La Princesa, 28006 Madrid, Spain; anablasu@hotmail.com (A.B.-S.); talarcon@helicobacterspain.com (T.A.-C.); 4 Department of Preventive Medicine, Public Health and Microbiology, School of Medicine, Universidad Autonoma de Madrid, 28049 Madrid, Spain

**Keywords:** grape seed extract, *Helicobacter pylori*, inflammation, oxidative stress, virulence

## Abstract

*Helicobacter pylori* (*H. pylori*) is a pathogenic bacteria identified as a potential risk factor for gastritis, gastric ulcers and gastric cancer. During the stomach colonization, *H. pylori* triggers a strong inflammatory response and subsequent oxidative stress, which are associated with tissue damage. For this reason, it is of particular interest to develop alternative natural tools that enable modulation of the associated damaging immune response. With this purpose, we obtained grape seed extract (GSE) from sweet (not fermented) food grade seeds. The aim of our study was to investigate the effect of GSE and its two enriched procyanidins fractions (OPC and PPC) on the inflammatory process and oxidative stress produced by different *H. pylori* strains in human gastric epithelial cells (AGS). Anti-inflammatory activity was evaluated by measuring the level of interleukin-8 (IL-8) secretion. IL-8 production was significantly reduced in *H. pylori*-infected human gastric epithelial cells pre-treated with GSE or its enriched fractions when compared with non-pre-treated infected cells (from 21.6% to 87.8%). Pre-treatment with GSE or its fractions significantly decreased intracellular reactive oxygen species (ROS) production in AGS cells after infection, depending on the *H. pylori* strain. Our results also showed that GSE and its fractions demonstrate antibacterial activity against all strains of *H. pylori* used in the study. This work demonstrates the effectiveness of GSE enriched in procyanidins against the main events associated with *H. pylori* infection.

## 1. Introduction

*Helicobacter pylori* (*H. pylori*) affects approximately 50% of the world’s population and the infection is acquired mainly during childhood. Unless an eradicating treatment is carried out, the infection may remain for life. Although the gastric colonization by *H. pylori* occurs asymptomatically in most people, long-term infection with this pathogen can cause a wide range of clinical manifestations that may progress from chronic active gastritis to peptic ulceration and gastric cancer [1,2,3]. In addition to gastric pathology related to *H. pylori* infection, this bacterium is associated with several extra-gastric pathologies, including, among others, cardiovascular, dermatological, autoimmune and hematologic diseases [4]. Also in the digestive system, *H. pylori* infection can lead to the development of extra-gastric pathologies, such as extra-gastric mucosa-associated lymphoid tissue (MALT)-lymphoma [5], gallstones [6], non-alcoholic fatty liver disease [7], hepatocellular carcinoma [8], and acute pancreatitis [9]. In 1994, *H. pylori* was classified as a type I carcinogen by the International Agency for Research on Cancer (IARC) [10] and it is the most important infectious cause of cancer worldwide, accounting for 810,000 attributable cases of cancer in 2018 [11]. Once *H. pylori* has reached the host stomach, it needs to survive in the acidic conditions, move around throughout flagella, and bind to gastric mucosal cells through the Outer Membrane Proteins (OMPs) [12]. *H. pylori* is able to adjust its periplasmic pH to about 6.1 in the extremely acidic environment of the stomach due to the enzyme urease, referred to as “acid adaptation”. It is encoded in a 7-gene cluster that includes catalytic units (*ure*A/B), an acid-dependent urea channel (*ure*I) and accessory structural urease apoproteins (*ure*EFGH). *H. pylori* urease is a holoenzyme that requires nickel as a cofactor and its uptake process involves the metallochaperones UreE and HypA [13]. *H. pylori* has between 4 and 7 polar flagella that allow it to move from the epithelial layer of the gastric mucosa to the basal layer, where the pH is close to seven. Studies in animal models have shown that the existence of flagella is essential for colonization and therefore *H. pylori* flagella are considered an early virulence factor [14]. Among the OMPs, the Blood Group Antigen-Binding Adhesion (BabA) is the best-characterized adhesin of *H. pylori*. Of the two alleles of *bab*A (*bab*A1and *bab*A2), only the product of *bab*A2 has the ability to bind fucosylated Lewis^b^ antigens [15]. The pro-inflammatory protein OipA is encoded by the *oip*A gene and correlates with interleukin-8 (IL-8) levels in the gastric mucosa [16]. It appears in 97.5% of patients with gastric or duodenal ulcers and in 70% of chronic gastritis patients [17].

In the process of stomach colonization, *H. pylori* triggers a strong inflammatory response mediated by neutrophils and macrophages, which generate the production of reactive oxygen species (ROS) by the epithelial tissue [18]. In addition to initial colonization of the stomach, *H. pylori* needs to persist in this environment and evade the host’s immune system. This process involves a number of virulence attributes of the pathogen, including proteins such as VacA and CagA, which are the most well-known. VacA is an important pore-forming cytotoxin. It is highly immunogenic and its main activity is to promote vacuolization in epithelial cells [19]. *H. pylori* have different allelic forms of the *vac*A gene that result in different classes of toxins. Although all strains contain the *vac*A gene, vacuolization in epithelial cells is only evoked by approximately 50% of strains. The expression of this protein plays an important role in the pathogenesis of peptic ulcers and gastric cancer [20]. *cag*PAI is a chromosomal region consisting of up to 32 genes encoding a multicomponent Type IV Secretion System (T4SS) and an effector protein, CagA. CagA is injected into cells through the *cag*T4SS, together with peptidoglycan peptides. This process causes an important cellular dysregulation and contributes to the induction of a strong inflammatory response, triggering the secretion of pro-inflammatory cytokines and chemokines, mainly IL-8. IL-8 represents a major marker of the inflammatory response of human epithelial cells to infection by *H. pylori* strains with a functional T4SS [21].

The group of events that comprise the immune response against *H. pylori* may be both beneficial and detrimental to the host according to their intensity. A strong immune response usually is not enough to remove the infection, and it can even provoke pathological damages in the host [22]. For this reason, it is of particular interest to have alternative treatments that allow the modulation of the immune response, thus avoiding the damage associated with the inflammatory process and subsequent oxidative stress. In this regard, there is a growing interest in the use of bioactive compounds from food components as natural alternative options to current treatments [23]. Among these compounds, grape seed extract (GSE) is one of the main known sources of catechins and procyanidins, since it is quite available and inexpensive, and may be potentially beneficial to human health. GSE has been shown to possess a number of bioactive properties such as antioxidant, anti-inflammatory and antimicrobial [24,25].

GSE has sometimes been shown to be effective as an antimicrobial agent against *H. pylori* [26], yet its effectiveness has not been demonstrated in other cases [27], probably due to differences in the composition of the extracts studied. However, although it is known that GSE could protect and provide an improved immunity response against chronic and acute gastric and intestinal oxidative injury [28], their impact on the inflammatory process and oxidative stress induced by *H. pylori* infection is scarcely known. This occurs mainly because the experimental models employed involve the use of chemical inductors of gastric damage [29], commercial lipopolysaccharide concentrates or other chemicals to induce the immune response in epithelial cells [30].

Recently, we demonstrated the antibacterial effect of GSE against several antibiotic resistant strains of *H. pylori*, also finding that procyanidins are the major phenolic component in the extract [31]. In the present work, we have investigated the effect of GSE and two fractions obtained from it—one enriched in oligomer procyanidins (OPC-rich), and the other enriched in highly polymerized procyanidins (PPC-rich)—on the inflammatory process and oxidative stress in the human gastric epithelial cell line AGS induced by different strains of *H. pylori,* in which their virulence attributes have been specially characterized. GSE was fractionated in order to establish a relation between bioactive properties against *H. pylori* and the degree of polymerization of the procyanidin chain.

## 2. Materials and Methods

### 2.1. Helicobacter pylori Strains, Growth Media, and Culture Conditions

*H. pylori* strains were isolated from gastric mucosal biopsies obtained from symptomatic patients from the Microbiology Department, Hospital Universitario La Princesa (Madrid, Spain). Biopsies were cultured in selective (*Pylori* agar, BioMerieux, Madrid, Spain) and non-selective media (Blood-supplemented Columbia Agar, BioMerieux, Madrid, Spain). *H. pylori* strains were stored at −80 °C in Brucella Broth (BB) (Becton, Dickinson, & Co., Madrid, Spain) with 20% glycerol. The agar-plating medium consisted of Müeller-Hinton agar supplemented with 5% defibrinated sheep blood (MHB) (Becton, Dickinson, & Co., Madrid, Spain), and the liquid growth medium consisted of BB supplemented with 10% horse serum (Biowest, Barcelona, Spain). *H. pylori* inoculum strains were prepared as follows: the frozen stored strains were reactivated by inoculation (200 μL) in an MHB plate and incubation in a microaerophilic atmosphere using a Variable Atmosphere Incubator (VAIN) (85% N_2_, 10% CO_2_, 5% O_2_) (MACS-VA500, Don Whitley Scientific, Bingley, UK) at 37 °C for 72 h. Bacterial biomass grown in one MHB plate was collected with a sterile cotton swab and suspended in 2 mL of BB or culture medium cell (~1 × 10^8^ colony forming units (CFU/mL)), and was then used as an experimental bacterial inoculum in the different experimental assays.

### 2.2. Determination of Antibiotic Susceptibility of H. pylori Strains

Antibiotic susceptibility of isolated *H. pylori* strains was performed by the E-test (BioMérieux, Madrid, Spain), defining the minimum inhibitory concentrations (MIC) against amoxicillin (AMX), clarithromycin (CLR), rifampicin (RIF), levofloxacin (LVX), tetracycline (TET), and metronidazole (MTZ). Bacterial inocula was prepared in BB supplemented with 10% horse serum, and 200 μL of this suspension was passed onto the surface of the MHB plates and streaked with a cotton swab. Antibiotic strips were placed onto the surface of inoculated MHB plates. For testing antibiotic susceptibility, the inoculated MHB plates were incubated in a microaerophilic incubator (VAIN) at 37 °C for 72 h before examination. MIC was determined taking the point where ellipse growth cut with the scale number in the E-test strip. The breakpoints were defined as follows: amoxicillin, MIC > 0.125 μg/mL; clarithromycin, MIC > 0.5 μg/mL; rifampicin, MIC > 1 μg/mL; levofloxacin, MIC > 1 μg/mL; tetracycline, MIC > 1 μg/mL; and metronidazole, MIC > 8 μg/mL; following the European Committee on Antimicrobial Susceptibility Testing (EUCAST) guidelines (version 8.0).

### 2.3. Identification of Virulence Markers in H. pylori Strains

For all the strains, acid nucleic extraction was performed from a 48 h *H. pylori* culture in blood agar using NucliSens^®^ easyMAG™ (Biomérieux, Madrid, Spain) according to the manufacturer’s instructions. An extract of nucleic acids was immediately frozen at −80 °C. Sequencing libraries were prepared with the TruSeq Nano DNA library prep kit (Illumina, San Diego, CA, USA) and were sequenced with an Illumina MiSeq instrument v3 chemistry generating paired-end reads of 300 bp each. The reads were trimmed using TrimGalore (https://www.bioinformatics.babraham.ac.uk/projects/trim_galore/, accessed on 23 April 2021) and assembled using SPAdes v.3.9.0 [32]. Annotation was performed using the Prokka pipeline v1.12 [33], using a curated annotation of the *H. pylori* 26695 genome as the primary annotation source. Presence or absence of virulence factors was determined according to the results of the previous annotation. Basic Local Alignment Search Tool (BLAST) from the National Center for Biotechnology Information (NCBI) was used to study the following genes and variants: allelic classification of *bab*A genes (*bab*A1 and *bab*A2 variants); allelic variants of *vac*A gene in the s, m, i, d, and c regions; and the presence of *ice*A and *dup*A genes. Alignments with sequence lengths similar to the gene size and identity percentage higher than 95% were accepted. The on/off status of the *oip*A gene was determined by analyzing the sequences as described by Sallas et al. [34] and by Dossumbekova et al. [35].

### 2.4. Preparation of the GSE and Its OPC-Rich and PPC-Rich Fractions

GSE was obtained through the maceration of sweet, food-grade grape seeds in 96% ethanol for 5 days at 40 °C as described by Silvan et al. [31]. The polymeric procyanidin-rich fraction (PPC) was separated from oligomeric procyanidins (OPC) by concentration/diafiltration with water on a 10 kDa molecular mass cut-off ultrafiltration membrane, as described by Gutierrez-Docio et al. [36]. Low molecular mass phenols that remained in the permeate stream were further purified from sugars, polyols, carboxylic acids, minerals and other non-dipole species by a preparative solid-phase extraction process on XAD7HP/XAD16 adsorbent resins. The OPC-rich fraction was recovered after desorption from the resin with ethanol [36]. GSE and its PPC-rich and OPC-rich fractions were freeze-dried and stored at 4 °C until use.

### 2.5. Human Gastric Epithelial Cell Cultures

The human gastric epithelial cell line AGS (gastric adenocarcinoma ATCC^®^ CRL-1739^TM^) was purchased from the American Type Culture Collection (ATCC, Barcelona, Spain). Cells were grown in Dulbecco’s Modified Eagle’s Medium/F12 (DMEM/F12) (Lonza, Madrid, Spain) supplemented with 10% fetal bovine serum (FBS) of South American origin (Hyclone, GE Healthcare, Logan, UK) and 1% penicillin/streptomycin (5000 U/mL) (Lonza, Madrid, Spain). Cells were plated at densities of ~1 × 10^6^ cells in 75 cm^2^ culture flasks (Sarstedt, Barcelona, Spain) and incubated at 37 °C under 5% CO_2_ in a humidified incubator until 90% confluence was reached. The culture medium was changed every two days. Before a confluent monolayer appeared, the sub-culturing cell process was carried out. All experiments were performed between passage 5 and 15 to ensure cell uniformity and reproducibility.

### 2.6. Evaluation of GSE Cytotoxicity

Before the cellular antioxidant and anti-inflammatory experiments, it was necessary to evaluate the cytotoxicity of GSE and its fractions against the AGS cell line. With this purpose, cell viability was determined by an MTT (3,4,5-dimethylthiazol-2,5-diphenyl-tetrazolium bromide) reduction assay, as was previously described by Silvan et al. [37]. Confluent cell cultures (~90%) were trypsinized (Trypsin/EDTA solution 170,000 U/L) (Lonza, Madrid, Spain) and cells were seeded (~5 × 10^4^ cells per well) in 96-well plates (Sarstedt, Barcelona, Spain) and incubated in a cell culture medium at 37 °C under 5% CO_2_ in a humidifier incubator for 24 h. Briefly, the cell culture medium was replaced with a serum-free culture medium containing the GSE and its fractions (2 mg/mL final concentration), and cells were incubated at 37 °C under 5% CO_2_ in a humidifier incubator for 24 h. Control cells (non-treated) were incubated in a serum-free culture medium without GSE and its fractions. Thereafter, cells were washed twice with phosphate-buffered saline (PBS) (Lonza, Madrid, Spain), and the medium was replaced with 200 μL of serum-free culture medium plus 20 μL of MTT (Merck, Sigma-Aldrich, Madrid, Spain) solution in PBS (5 mg/mL) that were added to each well for the quantification of the living, metabolically-active cells after 1 h incubation at 37 °C under 5% CO_2_ in a humidifier incubator. MTT is reduced to purple formazan in the mitochondria of living cells. Formazan crystals in the wells were solubilized in 200 μL of DMSO (Merck, Sigma-Aldrich, Madrid, Spain). After incubation, cell concentration was estimated as ranging from ~5 × 10^4^ to 5.5 × 10^4^ cells per well. Finally, absorbance was measured at 570 nm wavelengths, employing a microplate reader Synergy HT (BioTek Instruments Inc., Winooski, Vermont, USA). The viability was calculated considering controls containing the serum-free medium as 100% viable cells, and using the following formula:

Cell viability (%) = absorbance of sample/absorbance of control × 100

Data represent the mean and standard deviation (SD) of triplicates of three independent experiments (*n* = 9).

### 2.7. Study of the Effect of GSE and Its OPC-Rich and PPC-Rich Fractions on the Inflammatory Response Induced by H. pylori Strains in AGS Cells

Human gastric cells AGS were seeded (∼5 × 10^4^ cells/well) in 24-well plates (Sarstedt, Barcelona, Spain) and incubated in a cell culture medium at 37 °C under 5% CO_2_ in a humidifier incubator until a monolayer was formed. Cells were incubated with GSE and its fractions (1 mg/mL) at 37 °C in a 5% CO_2_ humidified atmosphere for 2 h. Cells were washed twice with PBS and infected with 0.5 mL/well of *H. pylori* inoculum prepared in a serum/antibiotics-free cell culture medium (∼1 × 10^8^ CFU/mL for all tested strains). The infected cells were incubated at 37 °C in a 5% CO_2_ humidified atmosphere for 24 h to allow the bacteria to adhere and invade the cells. Uninfected cells were included in the experiment as a control. At the end of incubation, supernatants from gastric epithelial cells were collected, particulate material was removed by centrifugation for 10 min at 12,000 rpm and samples were stored at −20 °C until analyses were performed. The amounts of secreted interleukin IL-8 in the collected supernatant of gastric epithelial cell samples were determined by an ELISA assay. A commercially available ELISA kit (Diaclone, Besancon, France) for the quantitation of IL-8 cytokine was used as described in the manufacturer’s instructions. The absorbance was measured at 450 nm using a microplate reader Synergy HT (BioTek Instruments Inc., Winooski, VT, USA). All the quantifications were performed in triplicate. Such as in the absence of bacteria, gastric cells release small amounts of IL-8 [38]; titers of cytokine released by AGS cells (pg/mL) were determined experimentally. Data represent the mean and SD of triplicates of three independent experiments (*n* = 9).

### 2.8. Determination of Antioxidant Activity of GSE and Its OPC-rich and PPC-rich Fractions against Intracellular Reactive Oxygen Species (ROS) Production in AGS Cells

The human gastric epithelial cell line AGS was used for the evaluation of oxidative stress. Intracellular ROS were measured by the DCFH-DA (carboxy-20,70-dichloro-dihydrofluorescein diacetate) assay, as was previously reported by Martín et al. [39]. Cells were seeded (5 × 10^4^ cells per well) in 24-well plates (Sarstedt, Barcelona, Spain) and grown until they reached 70% confluence. Cells were pre-treated with GSE and its fractions (1 mg/mL) dissolved in a serum-free cell culture medium for 24 h. After that, cells were washed twice with PBS and incubated with 20 μM DCFH-DA (Merck, Sigma-Aldrich, Madrid, Spain) at 37 °C for 30 min. Next, the cells were washed twice with PBS to remove the unabsorbed probe, and were then treated with *H. pylori* inoculum strains suspended in a serum/antibiotics-free cell culture medium (~1 × 10^8^ CFU/mL). ROS production was immediately monitored for 180 min in a fluorescent microplate reader Synergy HT (BioTek Instruments Inc., Winooski, Vermont, USA) using λ_ex_ 485 nm and λ_em_ 530 nm. After incubation, cell concentration was estimated as ranging from ~5 × 10^5^ to 5.5 × 10^5^ cells per well. After being oxidized by intracellular oxidants, DCFH-DA changes to dichlorofluorescein (DCF) and emits fluorescence. Cells treated only with the *H. pylori* inoculum were used as oxidative control (100% of intracellular ROS production). All samples were analyzed in triplicate in three independent experiments (*n* = 9).

### 2.9. Determination of Antibacterial Activity of GSE and Its OPC-Rich and PPC-Rich Fractions against H. pylori Strains

The antibacterial activity of GSE and its fractions against *H. pylori* strains was evaluated following the procedure described by Silvan et al. [40]. Briefly, 1 mL of GSE and its fractions (2 mg/mL final concentration) was transferred into different flasks containing 4 mL of BB supplemented with 10% horse serum. A bacterial inoculum (50 μL of ~1 × 10^8^ CFU/mL) was inoculated into the flasks under aseptic conditions. The cultures were prepared in triplicate and incubated under stirring (150 rpm) in a microaerophilic atmosphere using a VAIN at 37 °C for 48 h. Growth controls were prepared by transferring 1 mL of sterile water to 4 mL of BB supplemented with 10% horse serum and 50 μL of bacterial inoculum. After incubation, serial decimal dilutions of cultures were prepared in saline solution (0.9% NaCl) and were plated (20 μL) onto fresh MHB agar and incubated in a microaerophilic atmosphere using a VAIN at 37 °C for 72 h. All samples were analyzed at least in triplicate (*n* = 3). The number of CFU was assessed after incubation. The results of the antibacterial activity were expressed as log CFU/mL.

### 2.10. Chemical Characterization of GSE and Its OPC-Rich and PPC-Rich Fractions

Total phenolic content was determined by the Folin–Ciocalteu assay [40]. The results were expressed as mg of gallic acid equivalents/100 g dry mass extract. The determination of total procyanidins (TPC) was carried out by the acid butanol assay [41]. The results were expressed as mg of cyanidin equivalents/100 g dry mass extract. Total carbohydrates (the sum of all individual monomeric and dimeric saccharides), polyols (sorbitol and mio-inositol), and all other species that presented the same molecular masses and eluted in the interval where monomeric and dimeric saccharides and polyols do were determined by GC-FID-MS of their trimethylsilylated oxime derivatives, following the method of Montilla et al. [42]. Total catechins (catechin, epicatechin, and epicatechin gallate), total oligomeric procyanidins (OPC) (from non-galloylated and galloylated dimers to hexamers), and polymeric procyanidins (PPC) (chromatographically non-separable procyanidins that elute at the end of the chromatogram as a singular peak) were determined semi-quantitatively by NP-HPLC-PAD as previously described by Gutierrez-Docio et al. [36]. All analyses were done in triplicate from three independent samples (*n* = 9).

### 2.11. Statistical Analysis

The results were reported as means ± SD. Significant differences among the data were estimated by applying analysis of variance (ANOVA). Tukey’s least significant differences (LSD) test was used to evaluate the significance of the analysis. Differences were considered significant at *p* < 0.05. All statistical tests were performed with IBM SPSS Statistics for Windows, Version 25.0 (IBM Corp., Armonk, NY, USA).

## 3. Results

### 3.1. Strains Characterization: Antibiotic Susceptibility

Prevalence of antibiotic resistance and MIC values for the *H. pylori* strains are shown in Table 1. Overall, the highest resistance pattern was observed for metronidazole (4/6 strains), whereas no resistant strains were detected for tetracycline (0/6). Resistance to clarithromycin, amoxicillin, rifampicin, and levofloxacin was found in 3/6, 2/6, 2/6, and 1/6 of isolate strains, respectively. The most resistant strain was Hp58 (4/6), with resistance to amoxicillin, clarithromycin, levofloxacin, and metronidazole. Three strains were multiresistant (resistant to at least three or more classes of antibiotics: Hp58 in 4/6, and Hp53 and Hp61 in 3/6), all of them being resistant to clarithromycin. Two strains (Hp48 and Hp59) were found to be resistant to a single antibiotic (metronidaloze in both cases). Finally, only the strain Hp44 was susceptible to all antibiotics tested.

### 3.2. Characterization of Strains: Virulence Markers

All strains presented *ure*A, *ure*B, *ure*E, *ure*F, *ure*G, *ure*H, and *ure*I, as well as the metallochaperone *hyp*A. Likewise, studied genes related to flagellar function and structure (*fla*A, *fla*B, *fla*E, *rpo*N, *rpo*D, *flg*E, *flg*K, *flg*L, *flg*M, *flg*R, *flg*S, *flh*A, *fli*A, *mot*A, and *mot*B) were found in all strains. Table 2 shows the main genes in which differences were observed among studied strains. In four strains (Hp44, Hp48, Hp59, and Hp61), we found an annotation for the *cag*A gene, a marker of the pathogenicity island *cag*PAI. Of these four *cag*A-positive strains, three presented the seven genes described as essential for the functioning of the type IV secretion system (*cag*T, *cag*X, *cag*V, *cag*M, *cag*3, *cag*Y, and *cag*C), while in strain Hp44, neither the *cag*X nor *cag*V genes were found.

The study of *vac*A gene polymorphisms according to the alleles present in the s, m, i, d and c regions showed that the s2m2i2d2c2 configuration, related to lower virulence, was detected in three of the six strains (Hp53, Hp58 and Hp61). In contrast, strain Hp59 was the only strain with the s1-m1-i1-d1-c1 configuration, related to higher virulence and the most severe clinical conditions [43]. The other two strains showed mixed allele configurations. The study of the presence or absence of OMPs in each strain showed that *bab*A was present in strains Hp44, Hp48, Hp59 and Hp61. From these, Hp44 and Hp59 presented the A1 allele, while Hp48 and Hp61 had the A2 allele. Their paralogous genes were detected respectively in Hp44, Hp53, Hp58 and Hp61 in the case of *bab*B, and only in strain Hp59 for *bab*C. *sab*B, an orthologous gene of *sab*A (identified in all strains), was found only in strains Hp44 and Hp59. Lastly, all the strains studied presented the *oip*A gene. The analysis of the gene status showed that it was active (on) for Hp44, Hp48, Hp59, and Hp61, and inactive for Hp53 and Hp58.

### 3.3. Study of the Effect of GSE and Its OPC-Rich and PPC-Rich Fractions on the Inflammatory Response Induced by H. pylori in AGS Cells

Antioxidant and anti-inflammatory assays in AGS cells infected with *H. pylori* were carried out after pre-treatment with GSE or its fractions used at a concentration of 2 mg/mL. This concentration did not significantly (*p* ≤ 0.05) affect cell viability at 24 h after treatment (data not shown). We investigated the production of several pro-inflammatory cytokines in AGS cells stimulated by different strains of *H. pylori* and observed that IL-8 was the cytokine with the highest response (data not shown), so it was selected as an experimental marker. We studied the level of IL-8 production induced by the different *H. pylori* strains in AGS cells, and then we evaluated the effect of GSE on IL-8 secretion in AGS-infected cells. As shown in Table 3, uninfected AGS cells produced a background level of IL-8 (148.3 ± 11.9 pg/mL).

*H. pylori* strains were capable of stimulating IL-8 production from AGS cells in a strain-dependent manner. IL-8 production by AGS cells was significantly (*p* < 0.05) increased in response to exposure to all tested *H. pylori* strains with respect to the uninfected cells (control), except when the infection was induced by the Hp58 strain (196.6 ± 11.7 pg/mL). In this case, although an increase in IL-8 production was observed with respect to the control cells, the difference was statistically insignificant (*p* > 0.05) (Table 3). Hp48 (1525.8 ± 174.4 pg/mL) and Hp59 (1478.1 ± 44.8 pg/mL) were the most pro-inflammatory strains, stimulating up to ten-fold the production of IL-8 with respect to uninfected AGS cells (148.3 ± 11.9 pg/mL). In all cases, IL-8 production was significantly (*p* ≤ 0.05) reduced in AGS cells treated with GSE when compared with non-treated *H. pylori*-infected cells. GSE reduced IL-8 secretion by between 54.2% and 79.6%. The analysis of the behavior of each of the fractions obtained showed that both reduce IL-8 secretion. The PPC colloidal fraction showed greater anti-inflammatory activity, reducing IL-8 secretion in a similar way to GSE (from 51.8% to 87.8%). Interestingly, when AGS cells were infected with the two most pro-inflammatory strains (Hp48 and Hp59), the PPC fraction reduced IL-8 production by between 83.3% and 87.8%. In contrast, the OPC fraction showed a lower capacity to decrease IL-8 production (from 21.6% to 71.7%).

### 3.4. Antioxidant Activity against Intracellular Reactive Oxygen Species (ROS) Production in AGS Cells

**Figure 1:**
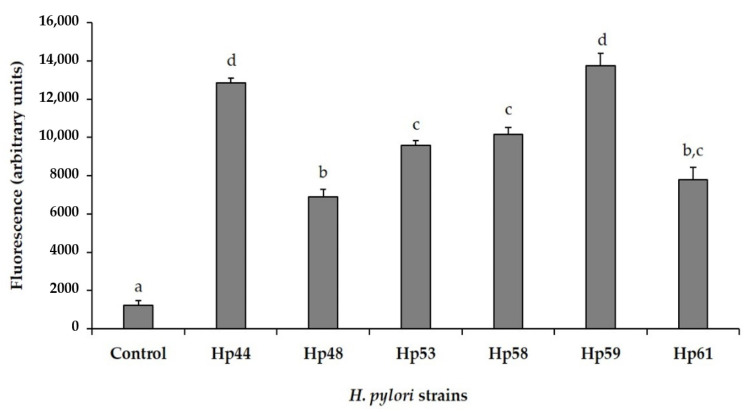
Induction of intracellular reactive oxygen species (ROS) production in human gastric AGS cells after exposure to *H. pylori* strains. ROS production was determined by fluorescence measurements. ^a, b, c, d^ values are expressed as fluorescence emission (mean value ± SD) (*n* = 9). Bars with different letters indicate significant differences in ROS production between control group (non-infected AGS cells) and *H. pylori*-infected cells with the different strains tested (Hp44, Hp48, Hp53, Hp58, Hp59, and Hp61) by ANOVA post hoc LSD Tukey test (*p* ≤ 0.05).

Infection of human gastric epithelial cell line AGS with *H. pylori* strains was associated with a rapid increase in fluorescence compared to levels of fluorescence measured in uninfected control cells (control), indicating the accumulation of intracellular ROS in *H. pylori*-infected cells (Figure 1). The patterns of intracellular ROS production was according to the strain tested, but all *H. pylori* strains were capable of inducing ROS generation in AGS cells. Strains Hp44 and Hp59 produced the highest amounts of intracellular ROS, while strains Hp48 and Hp61 induced the lowest amounts of oxidative damage in AGS cells.

**Figure 2:**
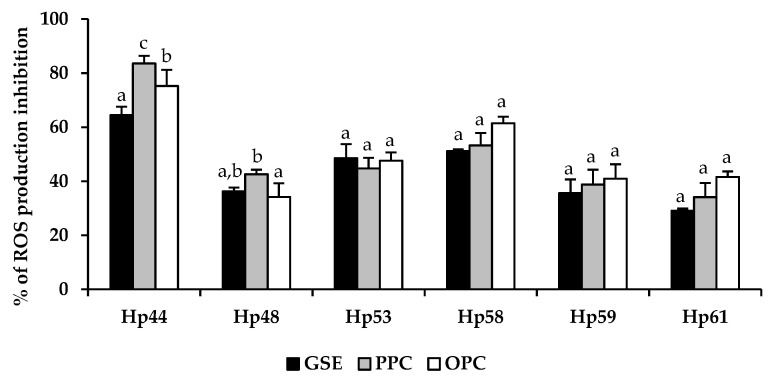
Protective effect of grape seed extract (GSE) and its polymeric procyanidin (PPC), and oligomeric procyanidin (OPC) fractions on intracellular reactive oxygen species (ROS) production. ^a, b, c^ values are expressed as inhibition of ROS production (%) (mean ± SD) with respect to the non-pre-treated control group (*n* = 9). Bars with different letters indicate significant differences between GSE and its fractions in the inhibition of ROS production for each *H. pylori* strain, determined by ANOVA post hoc LSD Tukey test (*p* ≤ 0.05).

As shown in Figure 2, in all cases, GSE significantly (*p* ≤ 0.05) decreased intracellular ROS production in AGS cells after infection with *H. pylori*. GSE induced an inhibition of ROS production between 30.1% and 71.0%, depending of the infective *H. pylori* strain. For most of the *H. pylori* strains, both fractions of GSE (PPC and OPC) contributed to the antioxidant effect in a similar way to the whole extract (GSE) (Hp53, Hp58, Hp59 and Hp61). For strains Hp44 and Hp48, a significantly greater effect (*p* > 0.05) of the PPC fraction was observed.

### 3.5. Antibacterial Activity

The antibacterial activity of GSE is presented in Table 4. Results showed different levels of growth inhibition. GSE at 2 mg/mL significantly (*p* ≤ 0.05) inhibited the growth of all tested *H. pylori* strains after 48 h of treatment. Reduction of log CFU/mL was from 2.87 to 5.79, depending on the *H. pylori* strain. MIC values in the presence of GSE were from 0.075 to 1.5 mg/mL. Although both fractions contributed to the antibacterial activity, PPC had the highest antibacterial activity. It showed a log reduction of CFU/mL ranging from 2.19 to 4.89, and MIC values from 0.075 to 0.1 mg/mL. In contrast, the activity of the OPC fraction was between 1.24 and 3.20 log CFU/mL, depending on the growth of the different *H. pylori* strains, with higher MICs than those for the PPC fraction.

### 3.6. Characterization of GSE and Its Fractions

The composition of GSE and its PPC-rich and OPC-rich fractions is shown in Table 5. The data of total phenolic (TPh) content indicate that both PPC and OPC fractions contained almost 1.4- and 2-fold higher amounts of phenolic compounds than the GSE, respectively. On the other hand, the total procyanidin content (TPC) shows that a higher enrichment of procyanidins was achieved in the PPC-rich fraction (1.7-fold) than the OPC-rich fraction (1.5-fold); however, in both cases, it was lower than 2-fold. This difference could be explained by the high sensitivity of the Folin–Ciocalteu assay to other grape seed constituents, such as reducing sugars. As higher amounts of sugars were present in the GSE (10.5 g/100 g), it should be considered that the value of 25.1 g/100 g for total phenols in the GSE could be somewhat overestimated.

**Figure 3:**
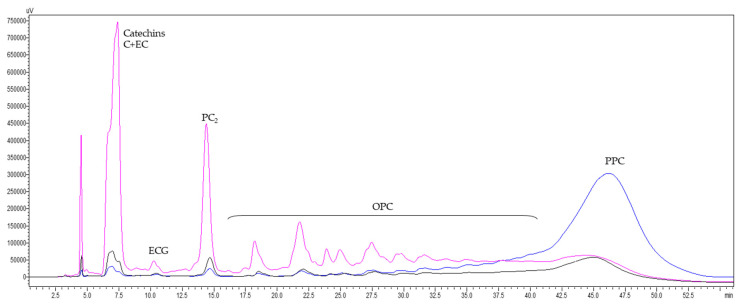
Overlay of NP-HPLC-PAD chromatograms of 20 mg/mL of grape seed extract (GSE) (black) and its oligomeric procyanidin (OPC) (violet) and polymeric procyanidin (PPC) (blue) fractions acquired at 280 nm. C+EC—catechin and epicatechin; ECG—epicatechin gallate; PC_2_—procyanidin dimers; OPC—oligomeric procyanidins; PPC—polymeric procyanidins.

To overcome the contradictions between the results from the two methods, a more specific trial for determination of procyanidins was carried out using NP-HPLC-PAD. This mode of analysis allows the depiction of procyanidin oligomers in an increasing order of molecular masses, as well as the separation of higher molecular mass polymers in a singular peak at the end of the chromatogram [36,44] (Figure 3).

**Figure 4:**
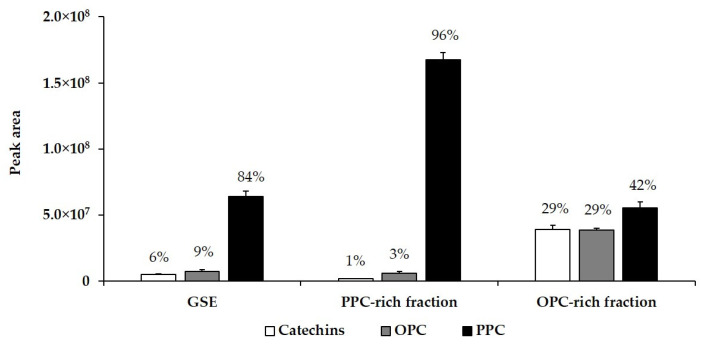
Sum of peak areas from chromatograms (Figure 3), corresponding to the sum of all catechins, total oligomeric procyanidin (OPC) and polymeric procyanidin (PPC) of the grape seed extract (GSE) and its fractions.

The results obtained for the sum of peak areas, corresponding to all catechins, OPC and PPC of the GSE are shown in Figure 4. They show that major compounds of GSE were PPC (84%), whereas catechins and OPC were at relatively low proportions of 6% and 9%, respectively. Separation of the macromolecular fraction allowed enrichment of PPC up to 96% of the total flavan-3-ol content, converting this fraction of highly purified PPCs. On the other hand, purification of the low molecular mass components allowed the recovery of a fraction enriched in catechins and monomeric and oligomeric (OPC) flavan-3-ols of up to 58% of the total flavan-3-ol content. Nevertheless, 42% of PPC remained in this fraction.

## 4. Discussion

In general, eradication therapy against *H. pylori* infection has been followed in symptomatic patients to date. People infected with *H. pylori* have a 3- to 6-fold higher risk of developing gastric adenocarcinoma or mucosa-associated lymphoid tissue (MALT) lymphoma than the rest of the population [45]. This suggests that all infected people should undergo eradication therapy. However, until now, eradication therapy against *H. pylori* has been mainly used in symptomatic patients. This is due, among other things, to the fact that the complete eradication of *H. pylori* may be associated with adverse effects, such as an increased risk of gastroesophageal reflux symptoms [45,46,47]. Eradication therapy is mainly based on the use of an antibiotic therapy. Currently, six antibiotics are mostly used: amoxicillin, clarithromycin, rifampicin, levofloxacin, tetracycline, and metronidazole, combined with proton pump inhibitors and/or bismuth salts. In this work, five of the six studied *H. pylori* strains showed antibiotic resistance. The highest level of resistance was observed for metronidazole (4/6) and clarithromycin (3/6) (Table 1). Metronidazole and clarithromycin-resistance rates are frequently reported, although they vary among different populations [48]. In the list of bacteria for which new antibiotics are urgently needed, clarithromycin-resistant *H. pylori* was included in the high priority group [49], and metronidazole has the most prevalent resistance pattern worldwide [50]. For other antibiotics that are usually very effective, such as amoxicillin, the appearance of some resistant strains (two o from six strains in this work) has also been observed, although in general, the level of reported resistance is very low in Europe (< 10%) [48]. Beyond antibiotic resistance, the presence of other bacterial virulence factors is significant for the pathogenesis and clinical course of *H. pylori* infection. Between virulence factors showing differences among strains, CagA has been classified as an oncogenic protein due to its association with gastric cancer [51]. It is reported in approximately 60% of *H. pylori* isolates [52,53], and we found it in four of the six strains (66%). Most studies on the prevalence of *cag*A are limited to studying the presence or absence of this gene, when in fact it is known that a large part of the world’s strains present rearrangements of the *cag*PAI island that render an inoperative T4SS [54]. In this work, it was found that three of the four *cag*A-positive strains presented the seven genes described as essential for T4SS functionality. In general, and considering the rest of the different virulence attributes all together, we can assume that the *H. pylori* strains selected for this work present a heterogeneous virulence profile that could be grouped, from highest to lowest, as follows: Hp59 > Hp48 > Hp61 > Hp44 > Hp53 = Hp58. Interestingly, *vac*A s2m2 strains (Hp53, Hp58, and Hp61), which could be considered to be less related to the progression of gastric damage, were resistant to clarithromycin, which has also been previously described by others [55,56].

The emergence of virulent *H. pylori* strains with antibiotic resistance has become a serious challenge all over the world. There is ample evidence on the influence of different virulence factors on a worse prognosis of the infection, and many of these virulence attributes are associated with a potent pro-inflammatory activity. However, on the other hand, it is thought that *H. pylori* usually contributes to modulating inflammation and limiting acute damage to the mucosa, enabling the bacteria to persist [57]. If this delicate balance is disturbed, disease may then develop. In this context, diet based treatments could contribute to mitigating the pro-inflammatory effects caused by *H. pylori* infection, without necessarily eradicating the bacterium [58]. With this aim, we have assessed the anti-inflammatory effect of GSE and its fractions. First, we observed that there are differences between the amount of IL-8 (the main cytokine released by gastric epithelial cells during gastric inflammation) that each strain of *H. pylori* is able to induce in AGS cells. The highest IL-8 levels were obtained for the two most virulent strains (Hp59 and Hp48), suggesting that there could be a relationship between virulence and the immune response. A similar behavior was previously observed in HT-29 intestinal cells infected with clinical strains of the closely related pathogen *C. jejuni* [37]. GSE caused a reduction of more than 50% in IL-8 production regardless of the infective strain. Phenols such as epicatechin gallate have shown strong inhibitory activity against IL-8 production [59]. IL-8 secretion is usually regulated by the transcription factor NF-kB, and *H. pylori* is known to induce IL-8 expression by activating the NF-k B pathway in gastric epithelial cells [30]. Since epicatechin gallate and some procyanidins present in GSE have been reported as NF-kB inhibitors [60,61,62], their suppressive effect on IL-8 secretion may correlate with their NF-k B inhibitory activity. Although there are compounds that participate in the observed effect for both PPC and OPC fractions, the highly polymerized PPC fraction appears to have a greater effect for most of the *H. pylori* strains.

The upregulation of IL-8 by *H. pylori* may lead to free-radical generation and the release of proteolytic enzymes from activated neutrophils, thus affecting mucosal integrity [57]. This is consequent to the induction of intracellular ROS production in AGS cells after exposure to *H. pilory* strains (Figure 2). The production of pro-inflammatory ROS as an essential defense mechanism against bacterial invasion can eventually become harmful for the host due to ROS-mediated tissue damage and inflammation [63]. In this study, GSE reduced intracellular ROS production in stomach epithelial cells, contributing to the modulation of epithelial damage. In most cases, the contributions of the two fractions (PPC and OPC) were very similar, suggesting that several phenolic compounds could be involved in the observed effect. Although chronic inflammation and ROS production are causative events associated with disease progression in gastric mucosa infected by *H. pylori*, it is difficult to predict in all cases if a reduction in ROS generation and release of pro-inflammatory IL-8 is the right choice, because they play an essential role in defense mechanisms against infection. The observed changes indicate inhibition of inflammation and may promote the progress of *H. pylori* infection. On the other hand, gastrointestinal mucosa exhibits a high cell proliferation rate similar to that seen in bone marrow [64]. For this reason, damage and/or loss of some gastric epithelial cells does not seem to be a high price to pay for the elimination of an infectious agent. Further research is needed to clarify this point. In any case, the investigations described here on ROS production and inflammation have been carried out using cell lines, which are often used to study biological processes and offer several advantages; they are cost effective, easy to use and able to provide consistent and reproducible results. However, these results are difficult to interpret and should be verified, at least in animal models. Previous studies have shown that retreatment with grapefruit seed extract, also rich in flavonoids, inhibits the development of experimental acute pancreatitis [65], and also protects gastric mucosa against damage evoked by ethanol or water immersion and restraint stress [66]. Intervention studies in humans should also allow us to evaluate the integration of the anti-inflammatory response with the nervous system and intestinal hormones, which play a relevant role in the physiological response associated with the gastrointestinal system. [67,68].

Finally, GSE acted as a potent antimicrobial agent against all *H. pylori* strains tested in a concentration range, which can be considered of practical interest for natural extracts [69]. The fraction enriched in high molecular weight procyanidins (PPC) showed the highest antimicrobial activity. We have previously described a similar behavior in a grape seed extract rich in procyanidins, where their structure facilitates the interaction between hydroxyl groups and the bacterial membrane [31]. This interaction would increase with the greater availability of hydroxyl groups related to the higher degree of polymerization of the procyanidin chains. The chemical characterization of the GSE showed that the extract consists mainly of polymeric procyanidins (84%). Concentration of this polymeric fraction (PPC) up to 96% (PPC rich fraction) led to an increase in the anti-inflammatory and antimicrobial capacity of the extract. It has been described that the degree of polymerization may play a determining role in the biological activity of procyanidins, although generalizations cannot be made because the size-activity relationship appears to be system dependent. However, in similar studies performed in models of colonic inflammation, it has been observed that high-molecular procyanidins polymers were most effective in reducing secretion of interleukin-8 in response to inflammatory stimuli [70]. PPCs have also been more effective in the protection of hepatic cells against cytotoxicity induced by oxidative stress, when compared to OPCs [71]. Further research is needed to characterize the role of grape seed PPCs in epidemiological and clinical intervention studies, which should contribute to the improvement of the preparation of bioactive extracts useful against *H. pylori*.

## 5. Conclusions

This study demonstrates the effectiveness of a grape seed extract enriched in procyanidins against the main events associated with *H. pylori* infection. GSE was able to significantly reduce inflammation and oxidative damage in *H. pylori*-infected stomach epithelial cells, and was also effective as an antimicrobial. This efficacy was observed independently of the degree of virulence and antibiotic resistance profile of the tested *H. pylori* strains. Both whole GSE and its fractions (PPC and OPC) showed activity against *H. pylori*. Enrichment of the extract in high molecular weight procyanidins (PPC fraction, higher than hexamers) apparently contributes to enhancing the bioactive properties of the extract against *H. pylori*. Further studies should attempt to clarify which of the two factors (larger polymer size and higher procyanidin content) is more relevant to the effect of the extract against *H. pylori*. This information could contribute to establishing new guidelines for a future food-based approach to *H. pylori* treatment.

## Figures and Tables

**Table 1:** Antibiotic resistance and MIC profile of *H. pylori* strains isolated from gastric biopsies.

Strains	Antibiotic Resistance (MIC) (mg/L)						Total Resistance
	AMX	CLR	RIF	LVX	TET	MTZ	
Hp44	S (0.023)	S (0.125)	S (0.38)	S (0.125)	S (0.125)	S (0.19)	0/6
Hp48	S (0.094)	S (<0.016)	S (0.5)	S (0.25)	S (0.25)	R (192)	1/6
Hp53	R (0.19)	R (4)	R (4)	S (0.125)	S (0.023)	S (0.75)	3/6
Hp58	R (1.5)	R (6)	S (0.75)	R (>32)	S (0.064)	R (96)	4/6
Hp59	S (0.023)	S (0.023)	S (1)	S (0.19)	S (0.125)	R (64)	1/6
Hp61	S (0.023)	R (12)	R (4)	S (0.38)	S (0.125)	R (>235)	3/6
Resistant strains	2/6	3/6	2/6	1/6	0/6	4/6	

AMX—amoxicillin; CLR—clarithromycin; RIF—rifampicin; LVX—levofloxacin; TET—tetracycline; MNZ—metronidazole. R—resistant; S—sensitive. MIC, Minimum Inhibitory Concentration.

**Table 2:** Virulence markers of *H. pylori* strains isolated from gastric biopsies.

Strains	*cag*A	*cag*PAI Essential Genes	*vac*A Alleles	*bab*A (Alleles)	*bab*B	*babC*	*sab*B	*oip*A (on)
Hp44	yes	No *cag*X, *cag*V	s1-m2-i1-d1-c2	yes (A1)	yes	no	yes	yes
Hp48	yes	yes	s1-m2-i2-d2-c2	yes (A2)	no	no	no	yes
Hp53	no	no	s2-m2-i2-d2-c2	no	yes	no	no	no
Hp58	no	no	s2-m2-i2-d2-c2	no	yes	no	no	no
Hp59	yes	yes	s1-m1-i1-d1-c1	yes (A1)	no	yes	yes	yes
Hp61	yes	yes	s2-m2-i2-d2-c2	yes (A2)	yes	no	no	yes

**Table 3:** Effect of grape seed extract (GSE) and its polymeric procyanidin (PPC), and oligomeric procyanidins (OPC) fractions on interleukin-8 (IL-8) production in human gastric AGS cells infected by *H. pylori* strains. IL-8 production was determined by ELISA assay. Values of IL-8 production are expressed as pg/mL (mean value ± SD) (*n* = 9).

Strains	Control	GSE	PPC Fraction	OPC Fraction
AGS cells	148.3 ± 11.9 ^A-a^	156.6 ± 5.3 ^a^	152.5 ± 10.1 ^a^	166.6 ± 8.4 ^a^
Hp44	841.6 ± 52.3 ^D-d^	295.8 ± 0.9 ^c^ (64.9%)	144.7 ± 11.8 ^a^ (82.8%)	239.2 ± 8.7 ^b^ (71.6%)
Hp48	1525.8 ± 174.4 ^E-c^	352.5 ± 37.0 ^b^ (76.9%)	255.3 ± 1.6 ^a^ (83.3%)	431.1 ± 10.7 ^b^ (71.7%)
Hp53	445.5 ± 40.5 ^C-c^	140.6 ± 1.2 ^a^ (68.4%)	149.4 ± 4.4 ^a^ (66.5%)	349.2 ± 0.9 ^b^ (21.6%)
Hp58	196.6 ± 11.7 ^A-b^	90.0 ± 6.7 ^a^ (54.2%)	94.7 ± 24.4 ^a^ (51.8%)	95.3 ± 11.1 ^a^ (51.5%)
Hp59	1478.1 ± 44.8 ^E-d^	300.8 ± 62.9 ^b^ (79.6%)	180.8 ± 26.0 ^a^ (87.8%)	510.8 ± 46.0 ^c^ (65.4%)
Hp61	345.8 ± 17.4 ^B,C-b^	127.5 ± 15.5 ^a^ (63.1%)	164.4 ± 22.5 ^a^ (52.5%)	149.2 ± 11.9 ^a^ (56.9%)

^A, B, C, D^ The same uppercase letter within the control column represents no significant difference (*p* > 0.05) between the strains by ANOVA post hoc LSD Tukey test. ^a, b, c, d^ The same lowercase letter within the same row represents no significant difference (*p* > 0.05) between treatments (GSE or its fractions) for each strain by ANOVA post hoc LSD Tukey test. Percentages shown in parentheses indicate the reduction in IL-8 production compared to the infected and untreated control.

**Table 4:** Effects of grape seed extract (GSE) and its polymeric procyanidin (PPC), and oligomeric procyanidin (OPC) fractions at 2 mg/mL on the viable counts of different *H. pylori* strains. Results of bacterial growth are expressed as log CFU/mL ± SD (*n* = 3).

Strains	Control Growth	GSE			PPC Fraction			OPC Fraction		
		2 mg/mL	log cfu/mL Reduction	MIC (mg/mL)	2 mg/mL	log CFU/mL Reduction	MIC (mg/mL)	2 mg/mL	log CFU/mL Reduction	MIC (mg/mL)
Hp44	7.85 ± 0.10 ^d^	4.98 ± 0.04 ^a^	2.87	0.075	5.66 ± 0.03 ^b^	2.19	0.075	5.97 ± 0.08 ^c^	1.88	0.25
Hp48	7.69 ± 0.06 ^d^	1.90 ± 0.35 ^a^	5.79	0.5	4.62 ± 0.07 ^b^	3.07	0.05	6.05 ± 0.01 ^c^	1.64	0.25
Hp53	7.44 ± 0.10 ^d^	2.16 ± 0.04 ^a^	5.28	0.075	3.88 ± 0.03 ^b^	3.56	0.05	4.84 ± 0.04 ^c^	2.60	0.05
Hp58	8.94 ± 0.11 ^d^	4.90 ± 0.07 ^b^	4.04	1.5	4.05 ± 0.02 ^a^	4.89	0.05	5.45 ± 0.07 ^c^	3.49	0.1
Hp59	7.73 ± 0.09 ^d^	3.94 ± 0.03 ^b^	3.79	0.5	3.38 ± 0.09 ^a^	4.35	0.1	6.49 ± 0.04 ^c^	1.24	0.5
Hp61	7.56 ± 0.04 ^c^	4.32 ± 0.09 ^b^	3.24	0.075	3.27 ± 0.20 ^a^	4.29	0.1	4.36 ± 0.03 ^b^	3.20	1.5

MIC: Minimum Inhibitory Concentration. ^a, b, c, d^ values of log CFU/mL in the same row marked with different letters indicate significant differences by ANOVA post hoc LSD Tukey test (*p* ≤ 0.05).

**Table 5:** Total phenolic, total procyanidin, and total carbohydrate contents of grape seed extract (GSE) and its polymeric procyanidin (PPC), and oligomeric procyanidin (OPC) fractions, expressed in g/100 g of dry mass.

**Analytical Parameters**	**GSE**	**PPC Fraction**	**OPC Fraction**
Total phenolic (TPh)	25.1 ± 0.5	34.9 ± 0.5	49.5 ± 1.3
Total procyanidin (TPC)	8.5 ± 0.3	14.6 ± 0.5	12.9 ± 0.4
Total carbohydrate (TCH)	10.5 ± 0.2	3.6 ± 0.2	0.36 ± 0.02

TPh content is expressed as equivalents of gallic acid; TPC content is expressed as equivalents of cyaniding; TCH is the sum of all monomer and dimer saccharides, and polyols obtained by GC-FID-MS.

## Data Availability

Data is contained within the article.
